# Study on the relationship between endometrial thickness on the hCG day in the progestin-primed ovarian stimulation protocol and pregnancy outcomes in frozen–thawed embryo transfer cycles

**DOI:** 10.3389/fendo.2025.1577872

**Published:** 2025-09-05

**Authors:** Yutong Wang, Yajie Chang, Shaohong Zhuang, Haitao Zeng, Xiaoyan Liang

**Affiliations:** ^1^ Reproductive Medicine Research Center, The Sixth Affiliated Hospital, Sun Yat-Sen University, Guangdong, Guangzhou, China; ^2^ GuangDong Engineering Technology Research Center of Fertility Preservation, Guangdong, Guangzhou, China; ^3^ Biomedical Innovation Center, The Sixth Affiliated Hospital, Sun Yat-Sen University, Guangdong, Guangzhou, China

**Keywords:** progestin-primed ovarian stimulation, endometrial thickness, frozen-thawed embryo transfer, pregnancy outcome, endometrial preparation

## Abstract

**Objective:**

The aim of this study was to investigate the relationship between endometrial thickness (EMT) on human chorionic gonadotropin (hCG) day in the progestin-primed ovarian stimulation (PPOS) protocol and pregnancy outcomes in the subsequent frozen–thawed embryo transfer (FET) cycles, providing new insights into embryo transfer strategies for patients undergoing the PPOS protocol.

**Methods:**

This retrospective study included 1,748 patients who underwent the PPOS protocol. Patients were divided into two groups based on the EMT on the hCG day: group A (EMT < 8 mm) and group B (EMT ≥ 8 mm). After 1:1 propensity score matching (group A: *n* = 701; group B: *n* = 701), the biochemical pregnancy, clinical pregnancy, and ongoing pregnancy rates were compared. In addition, the pregnancy outcomes under different endometrial preparation protocols were analyzed within each group.

**Results:**

(1) In groups A and B, the biochemical pregnancy rates were 44.5% and 45.5% (*p* = 0.707), the clinical pregnancy rates were 38.5% and 42.5% (*p* = 0.128), and the ongoing pregnancy rates were 29.1% and 34.2% (*p* = 0.039), respectively. For the cleavage-stage embryo transfers, groups A and B showed no significant differences in the biochemical pregnancy (39.5% *vs*. 35%, *p* = 0.285), clinical pregnancy (35.9% *vs*. 30.5%, *p* = 0.192), and ongoing pregnancy rates (26.8% *vs*. 24.4%, *p* = 0.527). For blastocyst transfers, the biochemical pregnancy (47.8% vs.51.2%, *p* = 0.307) was similar, but, clinical pregnancy (40.2% vs. 49%, *p* = 0.009), and ongoing pregnancy rates (30.6% vs. 39.6%, *p* = 0.005) were significantly higher in group B. (2) In group A, the endometrial preparation protocols had no statistically significant effect on the pregnancy outcomes. However, the natural cycles showed potentially better results (biochemical pregnancy rate, 43.1%; clinical pregnancy rate, 40.0%; and ongoing pregnancy rate, 34.1%) than hormone replacement therapy (HRT) (44.1%, 38.6%, and 29.9%, respectively), gonadotrophin-releasing hormone agonist plus HRT (GnRH-a+HRT) (45.1%, 39.3%, and 26.3%, respectively), and the mild stimulation cycles (27.8%, 16.7%, and 11.1%, respectively). In group B, patients using the GnRH-a+HRT protocol showed higher biochemical pregnancy (53.5%, *p* = 0.022), clinical pregnancy (48.7%, *p* = 0.096), and ongoing pregnancy rates (40.4%, *p* = 0.032) compared with those on natural cycles (38.2%, 33.9%, and 25.2%, respectively), HRT (42.9%, 40.8%, and 33.8%, respectively), and the mild stimulation protocol (45.5%, 45.5%, and 27.3%, respectively).

**Conclusions:**

The EMT on the hCG day in the PPOS cycle was positively related to the pregnancy outcomes of subsequent FET, with EMT ≥8 mm associated with better pregnancy outcomes, particularly in patients undergoing blastocyst transfers.

## Introduction

The progestin-primed ovarian stimulation (PPOS) protocol is a new controlled ovarian hyperstimulation (COH) protocol proposed by Professor Yanping Kuang in 2015 (1). In this protocol, prior to the increase of the estradiol (E2) levels in the follicle phase, exogenous progesterone is added to block the positive feedback effect of estrogen, while the negative feedback inhibits the secretion of luteinizing hormone (LH) from the pituitary gland. Both mechanisms work together to inhibit the early LH peak and to prevent premature ovulation. Current studies have confirmed that, for different populations with normal ovulation (2), polycystic ovary syndrome (3, 4), poor ovarian response (5), advanced age (6), or endometriosis (7), the PPOS protocol is safe and effective.

The endometrium is important for embryo implantation and development, the thickness and the morphology of which have a significant impact on the pregnancy outcomes (8). A thin endometrium during a fresh or frozen embryo transfer (ET) cycle impaired embryo implantation and development (9–11). Due to the negative effects of a supraphysiological dose of progesterone on endometrial receptivity, the PPOS protocol leads to frozen–thawed embryo transfer (FET) cycles. Therefore, no attention has been paid to the endometrial thickness (EMT) in the PPOS cycles and its correlation with the pregnancy outcomes of FET. This study aimed to investigate the EMT under PPOS protocol and its relationship with the subsequent pregnancy outcomes following FET in order to provide scientific evidence for the application and optimization of the PPOS protocol.

## Materials and methods

### Study population

This retrospective study was conducted from January 2015 to June 2019 in the Reproductive Medicine Research Center of the Sixth Affiliated Hospital of Sun Yat-sen University. The clinical data of the COH cycles and the first FET cycles were analyzed. The inclusion criteria were (1) age 20–50 years; (2) using PPOS protocol for COH and only using the embryos obtained from this cycle for FET. The exclusion criteria were (1) pre-implantation genetic test; (2) uterine abnormalities, including uterine adhesions, Cesarean scar diverticulum, and congenital uterine malformations (e.g., septate uterus, T-shaped uterus, unicornuate uterus, bicornuate uterus, etc.); (3) endometrial hyperplasia (with or without atypical hyperplasia); (4) polycystic ovary syndrome; and (5) use of clomiphene citrate (CC) and letrozole (LE) in COH.

This study is in line with the Declaration of Helsinki and has been approved by the Ethics Committee of the Sixth Affiliated Hospital of Sun Yat-sen University (2024ZSLYYEC-599).

### Clinical protocols

#### PPOS protocol for COH

Medroxyprogesterone acetate (MPA) (10 mg/day; Zhejiang Xianju Pharmaceutical Co., Taizhou, China), progesterone soft capsules (utrogestan, 200 mg/day; Laboratories Besins International, Paris, France), or dydrogesterone (Duphaston, 20 mg/day; Abbott Biologicals, Olst, the Netherlands) was administered from the third day of the menstrual cycle. At the same time, recombinant follicle-stimulating hormone (r-FSH), Puregon(Organon, Oss, the Netherlands) or Gonal-F (Ares Serono, Geneva, Switzerland), was administered at a starting dose of 100–300 IU/day according to age, body mass index, ovarian reserve, etc., until the trigger day.

The drug dose was adjusted according to the follicular development under transvaginal ultrasound and the serum FSH, LH, E2, and progesterone levels every 2–4 days. When one follicle reached a diameter of 18 mm or when two follicles reached a diameter of 17 mm, triptorelin (Decapeptyl, 0.1 mg; Ferring Pharmaceuticals, Kiel, Germany) and human chorionic gonadotropin (hCG) (6,000 to 10,000 IU; Lizhu Pharmaceutical Trading Co., Zhuhai, China) were administered as the trigger. The oocytes were retrieved 36 h later.

#### Embryo culture and embryo transfer

Under the guidance of transvaginal ultrasound, all follicles ≥10 mm in diameter were aspirated and *in vitro* fertilization (IVF) or intracytoplasmic sperm injection (ICSI), or a combination of IVF and rescue ICSI, was performed according to the semen parameters and the previous fertilization. Embryo culture was performed according to the routine of our center. For day 3 (D3) embryo evaluation, available D3 embryos were defined as embryos with more than four cells and <20% fragmentation or with four cells and no fragmentation. High-quality D3 embryos were defined as embryos with six to nine cells and <20% fragmentation. Blastocysts were graded using the Gardner system: ≥3CC was considered as available blastocysts, which could be frozen–thawed for ET, and ≥3BB was considered as high-quality blastocysts.

ETs were conducted within 3 months after COH. According to the status of the patient, natural cycles (NCs), hormone replacement therapy (HRT) cycles, gonadotrophin-releasing hormone agonist plus HRT (GnRH-a+HRT) cycles, or mild stimulation cycles were selected for endometrial preparation.

NCs were applied to patients with regular menstrual cycles. Monitoring of follicle development and serum LH levels was started from D10, and ET was conducted 3–5 days after ovulation.

Patients with irregular menstrual cycles or with a thin endometrium received HRT, with estradiol valerate from D3 (6–8 mg/day; Progynova, Bayer Healthcare Co., Berlin, Germany), and dydrogesterone (Duphaston, 20 mg, bid; Abbott Biologicals, Olst, the Netherlands) was added when the EMT was >8 mm. ET was performed 5–7 days after endometrial transformation.

Patients with endometriosis, adenomyosis, or poor pregnancy outcomes using other endometrial preparation protocols received the GnRH-a+HRT protocol. Triptorelin (Diphereline, 3.75 mg; Ipsen, Paris, France) was injected subcutaneously on D2–D3. After 28 days, when FSH < 5 IU/L, LH < 5 IU/L, and EMT < 5 mm, HRT and FET were performed as described above.

For patients with irregular menstrual cycles and luteal insufficiency, the mild stimulation protocol could also be used, with LE (2.5–5 mg/day; Jiangsu Hengrui Co., Lianyungang, China) or CC (50–100 mg/day; Fertilan, Codal Synto Ltd., Limassol, Cyprus) from D3 to D5. Follicle development and the EMT were monitored from D10. When necessary, human menopausal gonadotropin (hMG) was added to stimulate the growth of a single dominant follicle. ET was conducted 3–5 days after ovulation.

Luteal support was performed as routine: progesterone injection (40 mg/day; Zhejiang Xianju Pharmaceutical Co., Taizhou, China) intramuscularly combined with progesterone soft capsules (utrogestan, 200 mg/day; Laboratories Besins International, Paris, France) vaginally until D14 after transplantation.

### Outcome indexes

Serum β-human chorionic gonadotropin (β-hCG) was detected 12 or 14 days after ET. A β-hCG level ≥25 U/L was defined as a biochemical pregnancy. Clinical pregnancy was defined as the presence of one or more intrauterine gestational sacs and fetal heartbeats detected by transvaginal ultrasound 4–5 weeks after ET. An intrauterine clinical pregnancy confirmed by ultrasound again after 12 weeks of gestation was defined as an ongoing pregnancy.

### Statistical analysis

All statistical analyses were performed using IBM SPSS 27.0. Continuous variables were described as the mean and standard deviation (*x* ± *s*) and were calculated using Student’s *t*-test. Categorical variables were described according to the number of cases and the proportions, and the chi-square test was used.

In this study, 1:1 propensity score matching (PSM) was used, and the caliper was set to 0.02.

## Results

**Figure 1:**
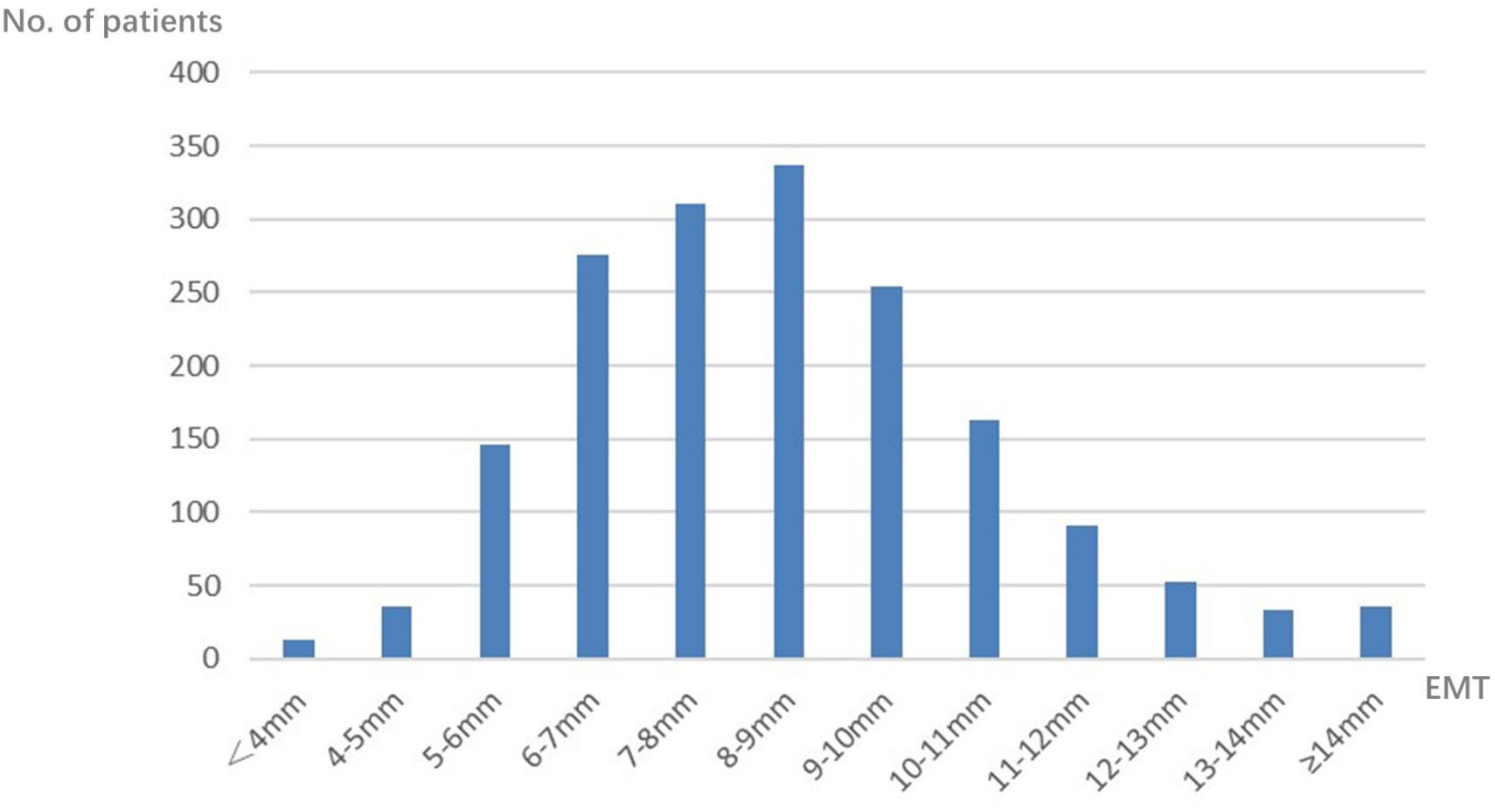
Distribution of endometrial thickness (EMT) on human chorionic gonadotropin (hCG) day in the progestin-primed ovarian stimulation (PPOS) cycles.

A total of 1,748 patients were included in this study, with EMT ranging from 1 to 18 mm (**Figure 1** for details). The mean EMT was 8.07 mm, and the median EMT was 8.00 mm. Therefore, 8 mm was selected as the cutoff value in this study.

### Baseline characteristics

According to the EMT in the PPOS cycles, the 1,748 patients were divided into two groups: group A, with EMT <8 mm on the hCG day (*n* = 782), and group B, with EMT ≥8 mm on the hCG day (*n* = 966). The univariate analysis indicated that there were significant differences in age, infertility type, anti-Müllerian hormone (AMH), the number of oocytes retrieved, the LH level on the hCG day, and the type of progesterone formulations between the two groups. To control for bias, a 1:1 PSM was performed between the two groups based on the variables with significant *p*-values in the univariate analysis. After matching, there were 701 patients in each group, with no statistically significant difference between the two groups (**Table 1**).

**Table 1:** Baseline characteristics of patients with different endometrial thickness (EMT) on human chorionic gonadotropin (hCG) day in the progestin-primed ovarian stimulation (PPOS) protocol.

Parameters	Before PSM			After PSM		
	Group A (*n* = 782)	Group B (*n* = 966)	*p*	Group A (*n* = 701)	Group B (*n* = 701)	*p*
Age (years)	35.99 ± 4.97	34.75 ± 5.20	<0.001	35.31 ± 4.72	35.25 ± 5.16	0.816
Infertility duration (years)	4.38 ± 3.59	4.57 ± 3.50	0.265	4.47 ± 3.60	4.59 ± 3.58	0.535
Infertility type			<0.001			0.665
Primary infertility	38.7% (304/782)	48.5% (471/966)		41.7% (292/701)	42.8% (300/701)	
Secondary infertility	61.3% (479/782)	51.5% (495/966)		58.3% (409/701)	57.2% (401/701)	
BMI (kg/m^2^)	22.27 ± 2.83	22.08 ± 2.93	0.181	22.21 ± 2.88	22.08 ± 2.90	0.397
AMH (ng/ml)	2.05 ± 2.09	2.50 ± 2.23	<0.001	2.12 ± 2.06	2.30 ± 2.03	0.087
Type of progestogen			0.003			0.105
MPA	51.2% (400/782)	43.3% (418/966)		52.8% (370/701)	48.9% (343/701)	
Utrogestan	35.7% (279/782)	43% (415/966)		35.7% (250/701)	41.1% (288/701)	
Dydrogesterone	13.2% (113/782)	13.8% (162/966)		11.6% (81/701)	10% (70/701)	
E2 on the hCG day (ng/L)			0.543			0.529
E2 < 1,000	30.4% (238/782)	29.0% (280/966)		29.1% (204/701)	31.1% (218/701)	
1,000 ≤ E2 < 2,000	32.6% (255/782)	29.5% (285/966)		32.2% (226/701)	28.7% (201/701)	
2,000 ≤ E2 < 3,000	17.8% (139/782)	19.8% (191/966)		18.1% (127/701)	20% (140/701)	
3,000 ≤ E2 < 4,000	6.0%% (47/782)	7.5% (72/966)		6.1% (43/701)	7.1% (34/701)	
4,000 ≤ E2 < 5,000	3.8% (30/782)	4.7% (45/966)		4.3% (30/701)	4.9% (34/701)	
5,000 ≤ E2 < 6,000	2.3% (18/782)	2.8% (27/966)		2.4% (17/701)	2.4% (17/701)	
E2 ≥ 6,000	7.0% (55/782)	6.8% (66/966)		7.7% (54/701)	5.8% (41/701)	
LH on the hCG day (U/L)	4.25 ± 2.90	3.89 ± 2.81	0.011	4.06 ± 2.82	4.06 ± 2.91	0.991
No. of oocytes retrieved			<0.001			0.983
Oocytes <5	25.4% (199/782)	17.9% (173/966)		21.3% (149/701)	20.5% (144/701)	
5 ≤ Oocytes < 10	46.4% (363/782)	46.6% (450/966)		43.8% (307/701)	44.7% (313/701)	
10 ≤ Oocytes < 115	16.76% (130/782)	19.4% (187/966)		17.7% (124/701)	17.8% (125/701)	
Oocytes ≥15	11.5% (90/782)	16.1% (156/966)		17% (119/701)	17% (119/701)	

Group A: Endometrial thickness (EMT) <8 mm on the human chorionic gonadotropin (hCG) day. Group B: EMT ≥8 mm on the hCG day.

PSM, propensity score matching; AMH, anti-Müllerian hormone; BMI, body mass index; MPA, medroxyprogesterone acetate; E2, estradiol; and LH, luteinizing hormone.

### Pregnancy outcomes

After 1:1 PSM, there were no statistical differences in the types of embryos transferred, the number of embryos transferred, and whether high-quality embryos were transferred. However, there were statistical differences in the EMT on the day of endometrial transformation between the two groups (**Table 2**). The biochemical pregnancy rate (44.5% *vs*. 45.5%, *p* = 0.707) and the clinical pregnancy rate (38.5% *vs*. 42.5%, *p* = 0.128) in group B were higher than those in group A, but there was no statistical difference, while the ongoing pregnancy rate (29.1% *vs*. 34.2%, *p* = 0.039) was statistically different. These results indicated that the patients in group B had better pregnancy outcomes after FET than the patients in group A (**Table 3**).

**Table 2:** Comparison of the frozen–thawed embryo transfer (FET) characteristics between the two groups after propensity score matching (PSM).

Parameters	Group A (*n* = 701)	Group B (*n* = 701)	*p*
Type of embryos transferred			0.097
Cleavage-stage embryo	39.4% (276/701)	35.1% (246/701)	
Blastocyst	60.6% (425/701)	64.9% (455/701)	
No. of embryos transferred			0.693
1	48.9% (343/701)	51.2% (359/701)	
2	51.1% (358/701)	48.8% (342/701)	
Transfer of high-quality embryos^a^			0.586
Yes	74.0% (519/701)	72.8% (510/701)	
No	26.0% (182/701)	27.2% (191/701)	
Endometrial preparation protocol			0.602
NC	19.3% (135/701)	18.7% (131/701)	
HRT	46.2% (324/701)	47.2% (331/701)	
GhRH-a+HRT	32.0% (224/701)	32.5% (228/701)	
Mild stimulation	2.6% (18/701)	1.6% (11/701)	
EMT on the day of endometrial transformation (mm)	9.36	10.12	<0.001

Group A: Endometrial thickness (EMT) <8 mm on the human chorionic gonadotropin (hCG) day. Group B: EMT ≥8 mm on the hCG day.

NC, natural cycle; HRT, hormone replacement therapy; and GhRH-a+HRT, gonadotrophin-releasing hormone agonist plus HRT.

a Transfer of at least one high-quality embryo.

**Table 3:** Comparison of the pregnancy outcomes between the two groups after propensity score matching (PSM).

Outcomes	Group A (*n* = 701)	Group B (*n* = 701)	*p*
Biochemical pregnancy rate	44.5% (312/701)	45.5% (319/701)	0.707
Clinical pregnancy rate	38.5% (270/701)	42.5% (298/701)	0.128
Ongoing pregnancy rate	29.1% (204/701)	34.2% (240/701)	0.039

Group A: Endometrial thickness (EMT) <8 mm on the human chorionic gonadotropin (hCG) day. Group B: EMT ≥8 mm on the hCG day.

### Comparison of the pregnancy outcomes in patients with different types of embryo transfer

Binary logistic regression analysis was conducted to further explore the relationship between EMT and the subsequent FET pregnancy outcomes of the different types of embryos transferred. After adjustment, there were no significant differences in the biochemical pregnancy rate (OR = 0.854, 95%CI = 0.581–1.254, *p* = 0.420), the clinical pregnancy rate (OR = 0.803, 95%CI = 0.542–1.191, *p* = 0.420), and the ongoing pregnancy rate (OR = 0.908, 95%CI = 0.591–1.395, *p* = 0.659) between the patients with varied EMT on the hCG day of the PPOS cycles receiving cleavage-stage embryo transfer. For patients with blastocysts transferred, although no significant differences in the biochemical pregnancy rate (OR = 0.114, 95%CI = 0.846–1.466, *p* = 0.442), the clinical pregnancy rate (OR = 1.418, 95%CI = 1.076–1.869, *p* = 0.013), and the ongoing pregnancy rate (OR = 1.475, 95%CI = 1.100–1.977, *p* = 0.009) was significantly higher in those with EMT ≥8 mm on the hCG day of the PPOS cycles (**Table 4**).

**Table 4:** Relationship between endometrial thickness (EMT) and the subsequent frozen–thawed embryo transfer (FET) pregnancy outcomes of the different types of embryos transferred.

Group	Biochemical pregnancy rate		Clinical pregnancy rate		Ongoing pregnancy rate	
	OR (95%CI)	*p*	OR (95%CI)	*p*	OR (95%CI)	*p*
For cleavage embryo transfer		0.420		0.275		0.659
EMT <8 mm	1		1		1	
EMT ≥8 mm	0.854 (0.581–1.254)		0.803 (0.542–1.191)		0.908 (0.591–1.395)	
For blastocyst transfer		0.442		0.013		0.009
EMT <8 mm	1		1		1	
EMT ≥8 mm	1.114 (0.846–1.466)		1.418 (1.076–1.869)		1.475 (1.100–1.977)	

Group A1: The age, anti-Müllerian hormone (AMH), and the number and quality of embryos transferred were adjusted as confounding factors.

OR, odds ratio and 95%CI, 95% confidence interval.

### Effect of different progestogen and endometrial preparation protocols on the pregnancy outcomes

Of the progestogen commonly used in the PPOS protocol, the EMT on the hCG day was significantly thinner in patients using MPA than in those using Utrogestan and dydrogesterone (**Table 5**).

**Table 5:** Effect of different progestogens on the endometrial thickness (EMT) on the human chorionic gonadotropin (hCG) day in the progestin-primed ovarian stimulation (PPOS) cycles.

Type of progestogen	EMT (mm)	*p*
MPA (*n* = 713)a	7.68	0.004
Utrogestan (*n* = 538)b	8.09	
Dydrogesterone (*n* = 151)c	8.00	

a vs. b *p* < 0.001; a *vs*. c: *p* = 0.104; and b *vs*. c: *p* = 0.657.

After adjusting for confounding factors such as age, infertility type, AMH, and number of oocytes retrieved by multivariate logistic regression, no significant differences were found between the three progesterone formulations on the pregnancy outcomes (**Table 6**).

**Table 6:** Relationship between the different progestin protocols and the subsequent frozen–thawed embryo transfer (FET) outcomes in patients with varied endometrial thickness on the human chorionic gonadotropin (hCG) day.

Group	Biochemical pregnancy rate		Clinical pregnancy rate		Ongoing pregnancy rate	
	OR (95%CI)	*p*	OR (95%CI)	*p*	OR (95%CI)	*p*
Group A		0.709		0.533		0.829
MPA (*n* = 370)	1		1		1	
Utrogestan (*n* = 250)	1.153 (0.815–1.632)	0.422	1.108 (0.779–1.575)	0.569	1.124 (0.767–1.647)	0.549
Dydrogesterone (*n* = 81)	1.126 (0668–1.898)	0.656	0.954 (0.565–1.611)	0.861	1.096 (0.631–1.903)	0.745
Group B		0.891		0.422		0.656
MPA (*n* = 343)	1		1		1	
Utrogestan (*n* = 288)	0.978 (0.706–1.353)	0.320	0.928 (0.669–1.289)	0.658	0.886 (0.627–1.254)	0.496
Dydrogesterone (*n* = 70)	0.761 (0.444–1.304)	0.077	0.694 (0.402–1.198)	0.190	0.798 (0.454–1.403)	0.433

Group A: Endometrial thickness (EMT) <8 mm on the hCG day. Group B: EMT ≥8 mm on the hCG day.

MPA, medroxyprogesterone acetate; OR, odds ratio; and 95%CI, 95% confidence interval.

The same method was used for the analysis of the different endometrial preparation protocols. In patients with EMT <8 mm on the hCG day, the mild stimulation protocol showed the lowest biochemical pregnancy, clinical pregnancy, and ongoing pregnancy rates. The GnRH-a+HRT protocol also showed a lower ongoing pregnancy rate. In patients with EMT ≥8 mm on the hCG day, the GnRH-a+HRT protocol showed higher pregnancy outcomes; however, there were no statistically significant differences between the other three protocols (**Table 7**).

**Table 7:** Effects of the different endometrial preparation protocols on the pregnancy outcomes.

Group			Biochemical pregnancy rate			Clinical pregnancy rate			Ongoing pregnancy rate	
	EMT (mm)	%	OR (95%CI)	*p*	%	OR (95%CI)	*p*	%	OR (95%CI)	*p*
Group A				0.365			0.243			0.052
NC (*n* = 135)	9.59	46.7% (63/135)	1		40.0% (54/135)	1		34.1% (46/135)	1	
HRT (*n* = 324)	9.15	44.1% (143/324)	0.836 (0.541–1.291)	0.303	38.6% (125/324)	0.836 (0.541–1.291)	0.418	29.9% (97/324)	0.677 (0.426–1.075)	0.098
GnRH-a+HRT (*n* = 224)	9.62	45.1% (101/224)	0.866 (0.545–1.375)	0.432	39.3% (99/224)	0.866 (0.545–1.375)	0.542	26.3% (59/224)	0.570 (0.346–0.941)	0.028
Mild stimulation (*n* = 18)	8.36	27.8% (5/18)	0.257 (0.069–0.965)	0.093	16.7% (3/18)	0.257 (0.069–0.965)	0.044	11.1% (2/18)	0.188 (0.039–0.900)	0.036
Group B				0.029			0.126			0.079
NC (*n* = 131)	10.36	38.2% (50/131)	1		35.9% (47/131)	1		25.2% (33/131)	1	
HRT (*n* = 331)	9.9	42.9% (142/331)	1.062 (0.687–1.640)	0.811	40.8% (135/331)	1.062 (0.687–1.640)	0.788	33.8% (112/331)	1.275 (0.792–2.053)	0.317
GnRH-a+HRT (*n* = 228)	10.24	53.5% (122/228)	1.556 (0.966–2.455)	0.019	48.7% (111/228)	1.556 (0.966–2.455)	0.057	40.4% (92/228)	1.807 (1.103–2.962)	0.019
Mild stimulation (*n* = 11)	10.57	45.5% (5/11)	1.377 (0.379–4.998)	0.739	45.5% (5/11)	1.377 (0.379–4.998)	0.627	27.3% (3/11)	0.935 (0.216–4.049)	0.929

Group A: EMT <8 mm on the hCG day. Group B: EMT ≥8 mm on the hCG day.

EMT, endometrial thickness; OR, odds ratio; 95%CI, 95% confidence interval; NC, natural cycle; HRT, hormone replacement therapy; and GhRH-a+HRT, gonadotrophin-releasing hormone agonist plus HRT.

## Discussion

EMT has long been considered a crucial parameter for endometrial receptivity and a predictor of pregnancy outcomes in assisted reproduction. Currently, clinical attention is primarily directed toward EMT during the ET cycle, whether in fresh or frozen–thawed ET. Previous studies have shown that, in the ET cycles, a thin endometrium on the hCG day or the day of endometrial transformation has negative effects on embryo implantation and pregnancy, with a cutoff value ranging from 6 to 8 mm (12–16). Under conventional COH protocols and standard endometrial preparation regimens, although the hormone levels varied, the endometrium generally maintained physiological patterns, supporting the normal proliferative and secretory transformation of the endometrium. Although there is a lack of direct evidence linking EMT during the COH cycle to the pregnancy outcomes in subsequent FET cycles, it is generally believed that the endometrial growth capacity remains relatively stable when the hormonal regulation follows a physiological course. A thin endometrium, commonly defined as an EMT of less than 7–8 mm on the day of hCG or the day of endometrial transformation, is thus widely recognized as a risk factor for poor pregnancy outcomes.

The development and the morphology of the endometrium are regulated by a complex interplay between estrogen and progesterone. Estrogen promotes endometrial proliferation during the follicular phase, while progesterone induces secretory transformation during the luteal phase, establishing a receptive environment for embryo implantation. In the PPOS protocol, exogenous progestins are administered during the follicular phase to suppress premature LH surges, which differentiates this protocol from conventional antagonist or agonist regimens. Due to the early administration of supraphysiological doses of progestogen, the endometrium loses its normal transformation in the PPOS cycles. Therefore, no previous researchers have studied the characteristics of the endometrium in the PPOS cycles. However, the dosage of progestogens used in PPOS is insufficient to induce endometrial atrophy and does not completely inhibit endometrial proliferation. The endometrium retains partial responsiveness to endogenous estrogen and, therefore, remains capable of growth. In our study population, the EMT on the hCG trigger day during the PPOS cycles exhibited considerable inter-individual variability. A meta-analysis in 2021 (17) also suggested that the EMT in a PPOS cycle might even exceed that in a conventional COH cycle. These findings prompted us to further investigate the clinical significance of EMT in PPOS cycles. Since all embryos in the PPOS cycles are cryopreserved and transferred in subsequent FET cycles, we sought to determine whether the EMT on the hCG day could serve as a surrogate marker for endometrial receptivity or predict the pregnancy outcomes in the subsequent FET cycle. Interestingly, a positive association was observed between the EMT on the hCG day in the PPOS cycles and the pregnancy outcomes in FET, suggesting a potential predictive value. This correlation may reflect inherent endometrial sensitivity to estrogen. Patients with higher EMT on the hCG day likely exhibit greater estrogen responsiveness, resulting in a more robust endometrial proliferation not only in the PPOS cycles but also in the subsequent FET cycles. Based on the median EMT values in our cohort and previous literature, 8 mm was selected as the cutoff value for analysis. The results demonstrated that patients with EMT <8 mm on the hCG day in the PPOS cycles had significantly lower pregnancy rates in the subsequent FET cycles. It was worth noting that patients with varied EMT on the hCG day in the PPOS cycles showed no statistical difference in the E2 levels, which further indicated that the difference in EMT is related to sensitivity to estrogen. Therefore, in the subsequent FET cycles, patients with thicker endometrium were more sensitive to estrogen, leading to better proliferation and pregnancy outcomes.

Importantly, the association between EMT and pregnancy outcomes was more pronounced in patients undergoing blastocyst transfers compared with those receiving cleavage-stage embryos. This may be attributable to the superior developmental competence and the better synchrony of blastocysts with endometrial receptivity. In contrast, the impact of EMT was less evident in cleavage-stage ETs, potentially due to their limited developmental potential or less precise synchronization. A previous large clinical study had similar conclusions (18). Another study (19) also mentioned that, for a thin endometrium, the embryonic stage no longer affects the pregnancy outcomes. These studies all illustrated, from different perspectives, the relationship between EMT and the ET stages.

For patients with different EMTs during the PPOS cycles, the different endometrial preparation strategies were compared. It was found that the NC for endometrial preparation had the best pregnancy outcomes for patients with EMT <8 mm on the hCG day of the PPOS cycles. Previous studies have shown that GnRH-a+HRT for endometrial preparation can improve the pelvic and uterine immune microenvironment and endometrial receptivity, leading to its wide use in endometrial preparation for patients with endometriosis. However, for the normal population, the application of GnRH-a is controversial (20, 21). In this study, GnRH-a+HRT did obtain a thicker endometrium; however, compared with the NC, this protocol improved the pregnancy outcomes only in patients with EMT ≥8 mm on the hCG day of the PPOS cycles, while the adverse effects in patients with EMT <8 mm on the hCG day of the PPOS cycles did not improve. The reason for this result was unclear and may be due to GnRH-a affecting the expression of other endometrial receptor factors, which is an issue that requires further clinical research in the future. The mild stimulation protocol had also been associated with poor pregnancy outcomes in this population. CC and LE, which are commonly used in the protocols, both had anti-estrogen effects, which may have negative impacts on endometrial growth. Patients with EMT <8 mm on the hCG day of the PPOS cycles had significantly lower EMT when using CC or LE for endometrial preparation than the other protocols. The endometrial growth inhibition by CC or LE was noticeable in these patients, while the patients with EMT ≥8 mm on the hCG day of the PPOS cycles were not obviously affected. This may be due to patients with EMT <8 mm in the PPOS cycles having a lower estrogen sensitivity and being more sensitive to this inhibition, resulting in a poor endometrial growth and affecting endometrial receptivity. However, fewer patients used the mild stimulation protocol for endometrial preparation in this study population, which may have also affected the reliability of the study results.

Previous studies have shown that different types of progestogens such as MPA, progesterone capsules, and dydrogesterone could achieve similar pregnancy outcomes when used in the PPOS protocol (22–24). Our research also indicated this point. Although there were certain differences in the effects of the three progestogens on the endometrium, the average EMT was all close to or reached 8 mm, and it had no significant difference on the pregnancy outcomes.

The limitations of this study were as follows: this was a retrospective cohort study, and the selection of the endometrial preparation regimen was mainly dependent on the clinical experience and the personal choice of patients. Although confounding factors were corrected through PSM and multivariate logistic regression, selection bias could not be avoided. In follow-up clinical studies, we need to prospectively collect more clinical samples, extend the follow-up time, improve the follow-up quality, and analyze the cumulative live birth rate and obstetric outcomes of different groups so as to provide more comprehensive support and reference for the individualized application of the PPOS protocol.

## Conclusion

A greater EMT on the hCG day in the PPOS cycles was positively correlated with improved pregnancy outcomes following FET, with EMT ≥8 mm being particularly beneficial in cases of blastocyst transfer.

## Data Availability

The raw data supporting the conclusions of this article will be made available by the authors, without undue reservation.
